# Visualizing regional myocardial oxygenation changes with statistically optimal colormaps

**DOI:** 10.1186/1532-429X-11-S1-P276

**Published:** 2009-01-28

**Authors:** Sotirios A Tsaftaris, Richard Tang, Rachel Klein, Debiao Li, Rohan Dharmakumar

**Affiliations:** 1 Department of Electrical Engineering and Computer Science, Northwestern University, Evanston, IL USA; 2 Department of Radiology, Northwestern University, Chicago, IL USA

**Keywords:** Severe Stenosis, Coronary Artery Stenosis, Pharmacological Stress, Myocardial Signal, Canine Study

## Introduction

Blood-oxygen-level dependent (BOLD) MRI may be used for detecting myocardial oxygenation (MO) changes secondary to coronary artery stenosis. Under pharmacological stress, areas in the myocardium supplied by a stenotic coronary artery are hypointense relative to healthy regions. Visualizing these changes requires manual windowing. In this paper a method for automatic visualization of myocardial signal changes reflecting the regional variations in oxygenation is presented, using images obtained from a canine study under controlled conditions. The objective of this study is to overcome the rather subjective step of windowing by establishing an optimal colormap that permits visualization of statistical changes in signal intensities between healthy and pathological cases.

## Purpose

To facilitate the evaluation of myocardial BOLD images by automating the detection of regional abnormalities in MO under pharmacological stress in the presence of coronary artery stenosis.

## Methods

### Data acquisition

Breath-held and ECG-gated short-axis cardiac phase-resolved 2D SSFP-based myocardial BOLD images were acquired in two dogs under pharmacological stress with and without left-circumflex coronary artery stenosis in a Siemens 1.5 T scanner. Scan parameters: voxel size = 1.2 × 1.2 × 6 mm^3^; flip angle = 90°; TR/TE = 5.7/2.9 ms; 20 cardiac phases.

### Image processing algorithms

In order to have the greatest myocardial surface available for analysis, only the end systolic images of the two image series (adenosine without stenosis (AD) and adenosine with severe stenosis (SS)) were identified. A rectangular region of interest was chosen around the heart and a segmentation algorithm was utilized to isolate and segment the myocardium. The myocardial AD intensities (ADv) were collected and a student-T distribution was fitted by maximum likelihood estimation of its parameters (mean (m), variance (s), and degrees-of-freedom). An optimal pixel colormap was created by assigning red hues to signal values below the threshold *m-s* and yellow hues to larger values. The color-coded myocardial segments for both images were overlaid over the grayscale original images. All operations were performed in MATLAB.

## Results

**Figure 1:**
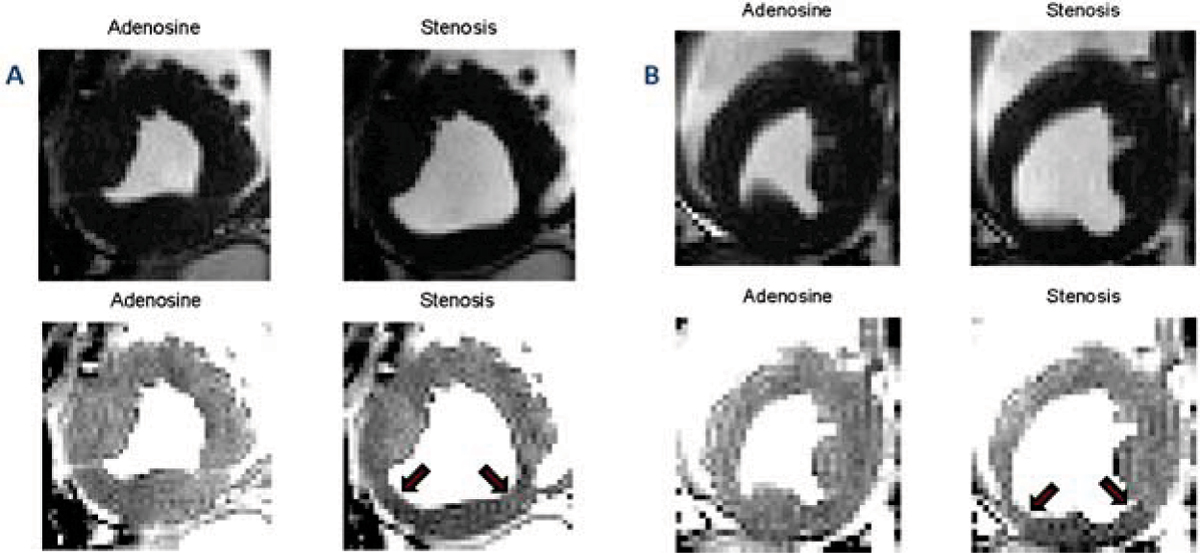
**End-systolic adenosine images without (AD) and with LCX stenosis (SS) from the two controlled canine studies are shown in panels (A) and (B), respectively**. The top row shows the AD and SS images without any windowing. The bottom row shows the same images but windowed the same to increase the regional myocardial contrast corresponding to oxygenation changes. Red arrows on the windowed SS images illustrate the limits of the affected myocardial segments corresponding to the LCX artery.

**Figure 2:**
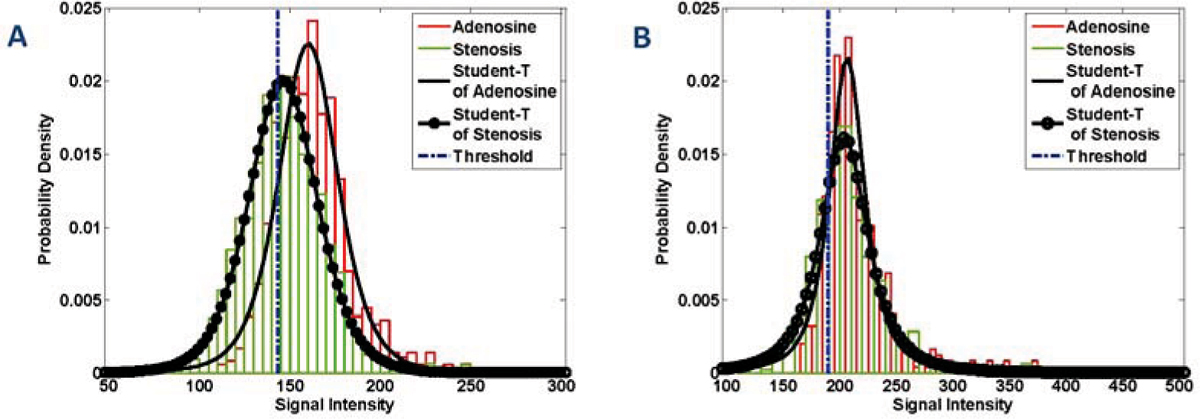
**Probability density plots for the two canine studies (A) and (B) are shown.** For each case the empirical densities of the adenosine and stenosis myocardial signals are plotted in red and green bars, respectively. Overlaid are the maximum likelihood estimated Student-T densities for each signal. Finally, the threshold is shown in blue at 143.3 for study A, 189.7 for study B, respectively. Note that m – s is the threshold computed from the Student-T distribution fitted to the adenosine signal as described in the text. All intensities below this threshold are color-coded in hues of red, while intesities above this threshold are color-coded in hues of yellow.

**Figure 3:**
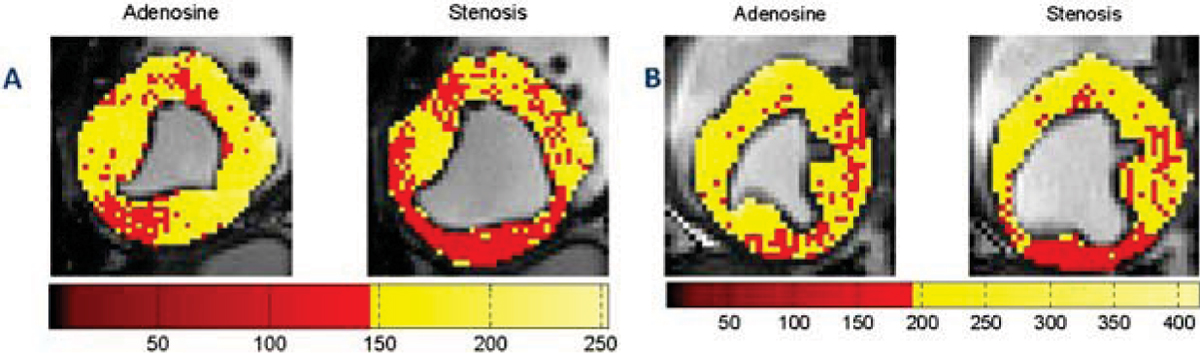
**Color-coded end-systolic adenosine images without (AD) and with LCX stenosis (SS) from the two controlled canine studies are shown in panels (A) and (B), respectively**. Only the myocardium was color coded. The segmentation masks were found using the algorithm in Tsaftaris, *et al.* ICIP 2008. The corresponding optimized colormaps are also shown. The yellow hues are for those values that are larger than the threshold as shown in Figure 2 and described in text. This method allows the observer to rapidly assess the presence of disease, as well as potentially identify the section of the coronary tree that is stenotic on the basis of BOLD MRI.

End-systolic AD and SS images from the two canine studies are shown in Figure 1 with, and without, windowing. The empirical distributions for (AD and SS) of the myocardial signal intensities and the corresponding fitted student-T distributions are shown in Figure 2. Figure 3 shows the color overlays utilizing the optimized colormap.

## Discussion and conclusion

As Figure 1 demonstrates, without proper windowing the appreciation of MO differences is cumbersome. It takes several minutes for a reader to window the images. Moreover, windowing is subjective and has large intra- and inter-observer variability. Figure 2 illustrates that student-T distribution closely approximates the intensity distribution of the myocardium. The motivation for choosing the *m-s* as the midpoint for the colormap is to highlight intensities below this value, which are expected to have low probability of occurrence in a normal healthy case. It is expected that regions supplied by stenotic arteries will be hypointense, and will thus shift the intensity distribution towards the left. Hence, in cases where stenosis is present, the occurrences of intensities lower than *m-s* are greater, and therefore the amount of red-colored pixels are greater. Note that the contiguous red-colored region corresponds to the left-circumflex territory (Fig. 3). There are also mild signal changes in the AD images as well, that may be due to imaging artifacts, normal physiology changes, and/or presence of an inadvertent stenosis. Nevertheless, the presented method allows the observer to rapidly assess the presence of disease, as well as, potentially identify the section of the coronary tree that is stenotic on the basis of BOLD MRI. The proposed method forms an initial step in the development of improved visualization capabilities for myocardial BOLD MRI. The method could be fully automated method if combined with end-systolic image identification and left ventricle localization algorithms.

